# Asymptomatic pharyngeal carriage rate of *Streptococcus pyogenes*, its associated factors and antibiotic susceptibility pattern among school children in Hawassa town, southern Ethiopia

**DOI:** 10.1186/s13104-019-4601-9

**Published:** 2019-09-10

**Authors:** Asrat Anja, Getenet Beyene, Zewdineh S/Mariam, Deresse Daka

**Affiliations:** 1 Dilla University College of Medicine, Dilla, Ethiopia; 2 Jimma University Institute of Health Sciences, Jimma, Ethiopia; 3 Hawassa University College of Medicine and Health Sciences, Hawassa, Ethiopia

**Keywords:** *S. pyogenes*, Nasopharyngeal carriage, Hawassa

## Abstract

**Objectives:**

The aim of this study was to determine the asymptomatic pharyngeal carriage rate of *S. pyogenes*, antimicrobial pattern and related risk factors among school children in Hawassa, southern Ethiopia.

**Results:**

Out of 287 school children’s screened, 35 (12.2%) were colonized with *S. pyogenes*. The carriage rate was significantly associated with factors such as sex (female p = 0.013) occupational status of mother (p = 0.002), lower income source (500–900 ETB, 1000–1500 ETB) (p = 0.001, and p = 0.042), history of hospitalization (p = 0.00) and residence of the children (p = 0.002). High level resistant to tetracycline and low level to vancomycin were observed, while penicillin, amoxicillin, erythromycin, chloramphenicol, and ceftriaxone were found to be effective.

## Introduction

*Streptococcus pyogenes* (*S. pyogenes*) is a species of gram positive, aerotolerant, beta-hemolytic bacterium in the genus Streptococcus. It is classified as Lancefield Group A Streptococcus (GAS). *S. pyogenes* has continued as a significant human pathogenic organism for centuries. It causes a several diseases in humans including mild skin disease, upper respiratory tract (URT) infections, severe life-threatening conditions such as Rheumatic fever, glomerulonephritis, septicemia, pneumonia and streptococcal toxic shock syndrome [1].

Infection begins with colonization of the URT or injured skin surfaces. All age group may carry GAS on throat and epidermis of skin, however, children aged 5–15 years old are a major reservoir of pharyngeal carriage of GAS [2].

GAS is highly communicable and can cause disease in individuals of all ages. School-age children (5–15 years) are considered as the major reservoir of GAS, with a prevalence of 2.5–25% or more depending on the study setting [3]. In Ethiopia, the asymptomatic carriage rate of GAS among healthy school children was 9.7–16.9% [4, 5].

An educational status, employment status of the family, separation of mother and father [6], sex [7–10], socioeconomic and environmental factors, lack of awareness of disease transmission are the risk factor for colonization of the *S. pyogenes* among school children [11, 12].

GAS has not been developed resistance to any of the penicillin’s over the last decades. Nowadays, it is starting to appear antimicrobial drug resistant strain from asymptomatic children [13] including penicillin [6]. However, in Ethiopia, antimicrobial resistance pattern to the GAS isolated from throat/phalanx were not adequately explicated. Therefore, the present study was aimed to determine the carriage rate of *S. pyogenes,* associated risk factors and antibiotic susceptibility pattern among school children in Hawassa, South Ethiopia, 2018.

## Main text

### Methods

#### Study area and period

Hawassa is the capital city of the Southern Nation Nationalities and people’s Government. It is located 275 km South of Addis Ababa, and has an altitude of 1665 m above sea level with mean annual temperature and rainfall of 20.9 °C and 997.6 mm, respectively.

#### Study design

A school based cross-sectional study design was conducted from May to October 2018 in Hawassa, South Ethiopia.

#### Source population

All school children who attached governmental primary schools found in Hawassa town during the study period.

#### Inclusion criteria

All children aged 5–15 years who attend the class in selected schools during the study period and children whose parents had accepted the consent to participate in the study.

#### Exclusion criteria

All children who were on antibiotics for the last 2 weeks those with any signs and symptoms of respiratory diseases such as fever, soreness and throat, cough and watery nasal discharge were excluded.

#### Study population

A total of 295 school children were enrolled in this study. The study participants were selected by using a multi-stage stratified sampling technique. From the total of 19 governmental primary schools 30% (5 of the schools) were included. Simple random sampling technique was used to select the schools. Proportionate amount of samples were assigned to each selected schools. Then study participants were identified by the lists of students using simple random sampling technique.

#### Specimen collection

The sample was collected from different school children from May to October 2018. The schools were sampled at the same period of time. All the swabs were immediately transferred to Amies transport medium (Oxoid, UK). Each sample was labeled very well. Within 2 h the collected swab were transported to the Microbiology laboratory [9, 14, 15].

#### Isolation of GAS

The throat swabs were directly inoculated to 5% sheep blood agar plates (Blood agar base, Oxoid UK) by rolling the swab over a small area of the plate and streaking the sample using a sterile loop and incubated at 37 °C with 5% CO_2_ atmosphere and examined beta-haemolytic colonies after 24 h and 48 h. Beta-haemolytic streptococci were identified by their colony morphology and beta-haemolysis.

All plates with beta-haemolytic colonies were sub-cultured with 0.04 U Bacitracin disks (Oxoid, UK) in blood agar plates similar manner as an earlier. All grams positive, catalase negative and any zone of inhibition around the disk were candidate for Pyrrolidonyl arylamidase (PYR) tests. A purple color in PYR tests were identified as *S. pyogenes* according to publications [16–18].

#### Antimicrobial susceptibility testing

Antibiotic susceptibility test (AST) was performed on disc diffusion method for all *S. pyogenes* using penicillin (10 U), erythromycin (15 µg), amoxicillin (10 µg), chloramphenicol (30 µg), ceftriaxone (30 µg), vancomycin (30 µg*)* and tetracycline (10 µg).

#### Data management and quality control

Quality of the data was ensured by using pre-structure questionnaire. For laboratory analysis the sterility of the prepared media was checked by incubating 5% by of prepared with in 5% CO_2_ enriched atmosphere at 37 °C for 24 h before using it. A quality control strain of *S. pyogenes* (ATCC19615) was used a positive control for each test.

#### Data processing and analysis

Data entry and analysis was performed by using SPSS version 20. The frequency of variables the prevalence of *S. pyogenes*, and antibiotic susceptibility pattern was determined. The association between risk factors and *S. pyogenes* colonization was determined by using logistic regression. A *p* value < 0.05 at 95% confidence interval (CI) was considered statistically significant.

### Result

#### Socio-demographic characteristics

Out of 295 school children who participated in the present study, 147 (51.2%) were males, 250 (87.8%) were within the age of 5–12 years. About 41.8% of the study participant’s parents/guardian found to have no formal education. A fifty percent of students’ mothers were a house-wife. About 162 (56.4%) of a total children’s parents/guardian had a monthly income between 500 and 900 ETB, 33 (11.5%) had 1000–1500 ETB and 92 (32.1%) had higher than 1500 ETB per month. However, the average income source of this area was 1560.00 ETB.

#### The prevalence of *S. pyogenes*

Among 287 school children 35 (12.2%) 95% CI [19–27.8] were confirmed to have *S. pyogenes* in throat swabs. A colonization rate of *S. pyogenes* among children who were 5–8 years old, 9–12 years old, 13–15 years old, those who live with employed mother, those who live with poor income source were 12 (17.1%), 18 (10.0%), 5 (13.5%), 8 (17.0%), and 26 (16%), respectively (Table 3).

The prevalence of *S. pyogenes* was higher among children with employed mother 8 (17.0%) than other occupations. Highest carriage rate was detected in low socioeconomic class 500–900 ETB per month 26 (16.0%) followed by 1000–1500 ETB 4 (12.1%). Among a total of 35 (12.2%) *S. pyogenes* isolates, the highest carriage rate was observed in student’s family size more than 5 person per house 23 (12.6%).

Out of 35 *S. pyogenes* isolated in this study, 35 (100%), 26 (74.3%), and 15 (42.9%) were susceptible to penicillin, vancomycin and tetracycline, respectively. About 34 (97.1%) of *S. pyogenes* isolates were sensitive to erythromycin, chloramphenicol, ceftriaxone and amoxicillin (Tables 1 and 2).

**Table 1:** Antimicrobial susceptibility pattern of *S. pyogens* isolated from school children at Hawassa city from May to October 2018 (n = 35)

Antimicrobial agents	Resistant n (%)	Susceptible n (%)
Penicillin	0 (0.0%)	35 (100%)
Vancomycin	9 (25.7%)	26 (74.3%)
Erythromycin	1 (2.9%)	34 (97.1%)
Chloramphenicol	1 (2.9%)	34 (97.1%)
Ceftriaxone	1 (2.9%)	34 (97.1%)
Amoxicillin	1 (2.9%)	34 (97.1%)
Tetracycline	20 (57.1%)	15 (42.9%)

**Table 2:** The predominant multiple antibiotic resistant phenotypes for *S. pyogenes* isolated from school children at Hawassa city from May–October 2018 (n = 24)

Phenotypes	Isolates tested N (%)
Tetracycline	13 (54.2)
Vancomycin	4 (16.7)
Tetracycline, amoxicillin	1 (4.2)
Tetracycline, ceftriaxone	1 (4.2)
Tetracycline, vancomycin	4 (16.7)
Tetracycline, vancomycin, erythromycin	1 (4.2)

#### Risk factor analysis for pharyngeal carriage

The possible risk factors such as age, sex, children living status, parents/guardians occupation, parents/guardians education, income of parents, family size, person per bed room sharing, and past history of recurrences of URTI were evaluated for pharyngeal carriage of *S. pyogenes.* It was observed in bivariate analysis that, female children (COR = 2.212; 95% CI 1.055–0.638; p = 0.013), low income of parents (COR = 3.326; 95% CI 1.231–8.990; p = 0.001), children being with mother(COR = 0.34; 95% CI 0.2–1.6; p = 0.301), and occupational status of mothers (COR = 1.8 (1.2–4.40) 95% CI 1.2–4.40; p = 0.02 were observed.

The female children (AOR = 2.730; 95% CI 1.24–6.037; p = 0.013), and low income of parents (AOR = 11.917; 95% CI 2.729–2.032; p = 0.001) were associated with *S. pyogenes* carriage. Conversely, Occupational status of mothers (AOR = 100; 95% CI 0.023–0.437; p = 0.002) was associated with reduced likelihood of risk for asymptomatic pharyngeal carriage of *S. pyogenes* (Table 3).

**Table 3:** Distribution and association of *S. pyogenes* among school children in Hawassa from May–October 2018

Variables	Total *S. pyogenes*		COR (95% CI)	Total N = 287 (100%)	p value
	Present n = 35 (12.2%)	Absent n = 252 (87.8%)			
Sex					
Female	23 (16.4)	117 (83.6)	1	140 (48.8)	*0.013*
Male	12 (8.2)	135 (91.8)	2.21 (1.8–3.14)	147 (51.2)	
Age					
5–8	12 (17.1)	58 (82.9)	0.76 (0.5–1.8	70 (24.4)	0.123
9–12	18 (10.0)	162 (90.0)	1.41 (0.8–3.20)	180 (62.7)	0.626
13–15	5 (13.5)	32 (86.5)	1	37 (12.9)	
Children living status					
With others^a^	7 (14.9)	40 (85.1)	0.64 (0.3–1.90)	47 (16.4)	0.427
Mother only	7 (25.0)	21 (75.0)	0.34 (0.2–1.6)	28 (9.8)	0.301
Father only	0 (0.0)	4 (100.0)	0	4 (1.4)	0.264
Both parent	21 (10.1)	187 (89.9)	1	208 (72.5)	
Educational status of parents					
Illiterate	19 (15.8)	101 (84.2)	0.89 (0.5–2.20)	120 (41.8)	0.365
1–4	1 (5.0)	19 (95.0)	3.2 (1.8–4.80)	20 (6.7)	0.309
5–12	10 (8.9)	102 (91.1)	1.7 (0.9–2.6)	112 (39.0)	0.546
> 12	5 (14.3)	30 (85.7)	1	35 (12.2)	
Occupational status of mothers					
Others^b^	10 (10.2)	88 (89.8)	1.8 (1.2–4.40)	98 (34.1)	*0.002*
House wife	17 (12.0)	125 (88.0)	1.5 (0.7–3.20)	142 (49.5)	*0.002*
Employed	8 (17.0)	39 (83.0)	1	47 (16.4)	
Occupational status of fathers					
Others^c^	21 (14.8)	121 (85.2)	0.35 (0.2–1.50)	142 (49.5)	0.446
Farmer	7 (10.8)	58 (89.2)	0.26 (0.18–1.94)	65 (22.6)	0.602
Employed	7 (8.8)	73 (91.2)	1	80 (27.9)	
Family income					
500–900	26 (16.0)	136 (84.0)	0.3 (0.2–1.68)	162 (56.4)	*0.001*
1000–1500	4 (12.1)	29 (87.9)	0.42 (0.3–2.20)	33 (11.5)	*0.042*
> 1500	5 (5.4)	87 (94.6)	1	92 (32)	
Past history of recurrent of URTI/RHD					
Yes	6 (14.6)	35 (85.4)	0.78 (0.6–1.70)	41 (14.3)	0.607
No	29 (11.8)	217 (88.2)	1	246 (85.7)	
Number of family per house					
< 5	12 (12.5)	84 (87.5)	1	96 (33.4)	0.208
≥ 5	23 (12.0)	168 (88.0)	10.9 (4.0–22.0)	191 (66.6)	
Residence					
Urban	16 (22.9)	54 (77.1)	1	70 (24.4)	
Rural	19 (8.8)	198 (91.2)	3.1 (1.7–5.8)	217 (75.6)	*0.002*
Cooking in bed room					
Yes	10 (7.0)	133 (93.0)	2.79 (1.2–6.4)	143 (49.8)	0.321
No	25 (17.4)	119 (82.6)	1	144 (50.2)	
Previous any disease					
Yes	25 (12.8)	170 (87.2)	0.83 (0.6–2.1)	195 (67.9)	
No	10 (10.9)	82 (89.1)	1	92 (32.1)	–
History of hospitalization					
Yes	22 (9.1)	220 (90.9)	4.1 (1.9–9.2)	44 (15.33)	*0.000*
No	13 (28.9)	32 (71.1)	1	45 (15.7)	

Italic values indicate significance of p value (p < 0.05)

*RHD* rheumatic heart disease, *AOR* adjusted odds ratio, *COR* crude odds ratio

^a^Guardians/care givers, ^b^ Both mother and father, ^c^ Daily labor, merchant, students

### Discussion

The overall asymptomatic pharyngeal carriage rate of *S. pyogenes* among school children was 12.2% which is higher than the reports in Ethiopia 9.7% [4], in Tunisia 9.0% [19], in Nigeria 10% [20], Pemba 8.6% [21], India 8.4% [12], and Mangalore 5% [7] and lower than the report of Ethiopia 16.9% [5], Turkey 13.9% [22], Pennsylvania 15.9% [11], Australia 19.5% [23] and Turkey 25.9% [15]. The possible explanation for the variation might be due to vaccination status and age differences. Moreover, sample size, seasonal variation and method if used, geography and socio-demographic variation are another possible explanation of the difference [4, 11, 21, 24].

In the present study we assessed different factors that could possibly increase colonization rate of *S. pyogenes*. Having female children 23 (16.4%) were 2.21 times more risk than male children for *S. pyogenes* colonization (p = 0.013). Similar result was reported from India [6], Turkey [4] and Nepal [5] and Ethiopia [4, 11, 21, 24]. This is might be due to social attitude towards female children or high contact with the others during supporting their mother in daily tasks.

The detection rate of *S. pyogenes* were high in children who had illiterate parents 19 (15.8%) (p > 0.05). It was in-lined with study reveled in India 44.9% [14], and in Iraqi 66.7% [25]. This might be reflects the literate parents had better awareness of the hygiene, hand washing and not sharing utensils than the illiterate parents.

There was clear association of pharyngeal carriage rate and children from low income families in our finding which 26 were (16.0%). It was in accordance with the earlier finding in Ethiopia [5] and other studies carried out in many parts of the world [7, 25].

The carrier group of *S. pyogenes* was higher 10.9 times more among children living within families of more than 5 members than less family members in our study (p > 0.05). Similarly, *S. pyogenes* carriage was significantly higher in large family size as reported in different authors (p < 0.05) [2, 23, 26]. This might be an increasing the number of family members increases the rate of prevalence of many infectious diseases including GAS.

A Children with a history of hospital admission had 4.1 times chance to be colonized with GAS (p = 0.00) which is comparable to other’s studies [27]. A children with history of recurrent pharyngitis show higher incidence 8 (80%) of carrier than those with no history of pharyngitis 16 (17.8%) [25].

A children from rural resident were 3 times (p = 0.002) more likely to have *S. pyogenes* carriage. This finding is similar to that the report found in Uganda at Wakiso district [28]. In the current study, a high proportion (12.8%) of previously diseased children was colonized with *S. pyogenes* even though it was not statistically significant (p > 0.05).

Out of 35 of *S. pyogenes* isolated in this study, 35 (100%), 26 (74.3%) and 15 (42.9%) were susceptible to penicillin, vancomycin and tetracycline, respectively. However, 34 (97.1%) of *S. pyogenes* were susceptible to erythromycin, ceftriaxone, chloramphenicol and amoxicillin. The finding is similar as compared with the report found in Ethiopia [4] and in different parts of the world [7–9, 12–14, 29–34]. About 7 (29.2%) of isolated GAS strains were showed multiple drug resistant (two or more drugs). This is might be due to the ease of availability of antibiotics and misuse of the drugs.

### Conclusion

The prevalence of *S. pyogenes* among school children in this study was high. The gender difference and low income of parents, residence of the children and occupational status of the mother are the prime risk factors associated with the carriage rate. All *S. pyogenes* isolates were susceptible to penicillin but most isolates were susceptible to chloramphenicol, Erythromycin and Ceftriaxone. Low level of resistant was observed against vancomycin and high level was observed against tetracycline. Further study in the area by using large sample size and all predisposing factors should be investigated.

## Limitation of the study

Serotyping of Group A streptococci and ASO titer was not performed due to lack of antisera.

## Data Availability

The data used/analyzed during the current study available from the corresponding author on reasonable request.

## Abbreviations

ETB: Ethiopian birr
ATCC: American type culture control
